# Robot-assisted laparoscopic radical cystectomy with intracorporeal ileal conduit diversion versus open radical cystectomy with ileal conduit for bladder cancer in an ERAS setup (BORARC): protocol for a single-centre, double-blinded, randomised feasibility study

**DOI:** 10.1186/s40814-022-01229-3

**Published:** 2023-01-13

**Authors:** Sophia Liff Maibom, Ulla Nordström Joensen, Eske Kvanner Aasvang, Malene Rohrsted, Peter Ole Thind, Per Bagi, Thomas Kistorp, Alicia Martin Poulsen, Lisbeth Nerstrøm Salling, Henrik Kehlet, Klaus Brasso, Martin Andreas Røder

**Affiliations:** 1 Urological Research Unit, Department of Urology, Faculty of Health and Medical Sciences, University of Copenhagen, Rigshospitalet, Copenhagen, Denmark; 2 Department of Anaesthesiology, Centre for Cancer and Organ Diseases, University of Copenhagen, Rigshospitalet, Copenhagen, Denmark; 3 Section of Surgical Pathophysiology, University of Copenhagen, Rigshospitalet, Copenhagen, Denmark

**Keywords:** Randomised controlled trial, Blinding, Radical cystectomy, Bladder cancer, Urothelial carcinoma, Robotic surgery, Open surgery

## Abstract

**Background:**

Radical cystectomy (RC) with urinary diversion is the recommended treatment for selected cases of non-metastatic high-risk non-muscle-invasive and muscle-invasive bladder cancer. It remains unknown whether robot-assisted laparoscopic cystectomy (RARC) offers any advantage in terms of safety compared to open cystectomy (ORC) in an Enhanced Recovery After Surgery (ERAS) setup. Blinded randomised controlled trials (RCTs) between RARC versus ORC have never been conducted in cystectomy patients. We will investigate the feasibility of conducting a double-blinded RCT comparing ORC with RARC with intra-corporal ileal conduit (iRARC) in an ERAS setup.

**Methods:**

This is a single-centre, double-blinded, randomised (1:1) clinical feasibility study for patients with non-metastatic high-risk non-muscle-invasive or muscle-invasive bladder cancer scheduled for cystectomy. All participants are recruited from Rigshospitalet, Denmark. The planned sample size is 50 participants to investigate whether blinding of the surgical technique is feasible. Participants and postoperative caring physicians and nurses are blinded using a pre-study designed abdominal dressing and blinding of the patient’s electronic health record. Study endpoints are assessed 90 days postoperatively. The primary aim is to study the frequency and pattern of unplanned unblinding after surgery and the number of participants who cannot guess the surgical technique at the day of discharge. Eleven secondary endpoints are assessed: length of stay, days alive and out of hospital, in-hospital complication rate, 30-day complication rate, 90-day complication rate, readmission rate, quality of life, blood loss, pain, rate of moderate/severe post-anaesthesia care unit (PACU) complications, and delirium. Participants are managed in an ERAS setup in both arms of the trial.

**Discussion:**

We report on the design and objectives of a novel experimental feasibility study investigating whether blinding of the surgical technique in cystectomy patients is possible. This information is essential for the design of future blinded trials comparing ORC to RARC. There is a continued need to compare RARC and ORC in terms of both efficacy, safety, and oncological outcomes. Estimated end of study is March 2021.

**Trial registration:**

ClinicalTrials.gov ID: NCT03977831. Registered on the 6th of June 2019.

**Supplementary Information:**

The online version contains supplementary material available at 10.1186/s40814-022-01229-3.

## Introduction

### Background and rationale

Radical cystectomy (RC) with urinary diversion for bladder cancer (BC) is the recommended treatment in selected cases of high-risk non-muscle-invasive BC and muscle-invasive BC [1]. The procedure is associated with a high risk of complications and often long recovery. There has been an increasing interest in introducing minimally invasive surgery in cystectomy patients to reduce complications and enhance recovery associated with RC. The introduction of robot-assisted laparoscopic RC (RARC) has led to several studies comparing RARC to standard open radical cystectomy (ORC) [2]. To date, there is no evidence that RARC reduces complications associated with RC compared to ORC [3].

Secondly, Enhanced Recovery After Surgery (ERAS) protocols have been promoted to reduce both complications and length of stay (LOS) associated with RC. ERAS protocols focus on improving and standardising perioperative care with an emphasis on evidence-based principles. ERAS protocols in RC have shown a decrease in complication rate and LOS without increasing the readmission rate, but it remains unknown if recovery is faster with RARC compared to ORC [4]. Currently, no RCTs, including blinding, comparing ORC and RARC in a contemporary perioperative ERAS setup have been published [5–9].

Blinding seeks to prevent ascertainment bias as care providers’ expectations may affect subjective outcomes [10]. Blinded RCTs in surgery are rare and has never previously to our knowledge been conducted for RC. We believe it is possible to conduct a blinded RCT in RC as blinded RCTs comparing open and laparoscopic procedures in abdominal surgery have been shown feasible [11–14].

This protocol describes an ongoing RCT assessing the feasibility of a double-blinded setup comparing ORC and robot-assisted laparoscopic RC with intracorporeal urinary diversion (iRARC) in an ERAS setup. The aim of the study was to test if blinding of the surgical technique is possible and secondly to describe efficacy and safety outcomes for the two surgical methods in an ERAS protocol.

## Methods/design

This study is designed as a randomised, controlled, patient, and care provider blinded single-centre feasibility study with 1:1 ratio allocation of participants to either ORC or iRARC. We will enrol a total of 50 participants. The clinical assumption is that the planned sample size is sufficient to explore the primary outcome and describe if blinding is feasible [15].

This trial is registered on the 6th of June 2019 at ClinicalTrials.gov ID: NCT03977831 (https://clinicaltrials.gov/ct2/show/NCT03977831?term=NCT03977831&draw=2&rank=1). The protocol version is the original from the 14th of December 2018: Protocol-60292_v1_141218. The protocol for this randomised trial is reported in compliance with the Standard Protocol Items Recommendations for Interventional Trials (SPIRIT) guidelines (see appendix for SPIRIT checklist) [16].

### Objectives

#### Primary objective

The primary objective of this study is to analyse the number of patients with unplanned unblinding before discharge and the number of patients who cannot infer the type of surgery.

#### Secondary objectives

The secondary objective of this study is to compare LOS, days-alive-and-out-of-hospital (DAOH), in-hospital complication rate, 30-day complication rate, 90-day complication rate, readmission rate, quality of life (European Organisation for Research and Treatment of Cancer (EORTC) Quality of Life Questionnaire QLQ-C30 and QLQ-BLM30), blood loss (estimated and hidden), pain, rate of moderate/severe post-anaesthesia care unit (PACU) complications (Danish Society of Anaesthesiology and Intensive Care (DASAIM) discharge criteria), and delirium in ORC versus iRARC.

### Study setting

All participants are recruited from and operated at Copenhagen University Hospital, Rigshospitalet. The Department of Urology, Rigshospitalet, is one of two tertiary referral centres for cystectomy in eastern Denmark.

Two highly experienced surgeons are assigned to perform iRARC and ORC (complete procedure), respectively. The surgeon who performs ORC has an experience of >500 ORCs. The surgeon who performs iRARC has performed >100 iRARCs. All procedures will be performed with one of three urology speciality registrars as assistants who are all experienced with both iRARC and ORC.

### Eligibility criteria and inclusion

#### Inclusion criteria

- High-risk non-muscle-invasive including treatment-resistant carcinoma in situ or muscle-invasive urothelial carcinoma of the bladder
- Age > 18 years
- Patient preference for an ileal conduit as urinary diversion
- Informed consent

#### Exclusion criteria

- Inability to speak/understand Danish
- Inability to cooperate for inclusion in the study
- Need for concomitant extended surgery (i.e., nephroureterectomy)
- Prior downstaging chemotherapy (neoadjuvant chemotherapy is allowed)
- Metastatic disease
- Prior pelvic radiation therapy
- Prior major extensive abdominal or pelvic surgery
- Prior peritonitis
- Conditions contraindicating Trendelenburg’s position.

Patients potentially eligible for the study are screened at the time of electronic referral. All bladder cancer patients are seen in the out-patient clinic every Thursday and one Friday per month. Pre-planned surgical slots for protocol patients are planned one month in advance. Eligibility is assessed in the outpatient clinic at the preoperative consultation. Patients will receive comprehensive oral information as well as written information material. Patients will be informed that their agreement to participate is voluntary. Hereafter, a second consultation is scheduled at which potential participants will have the opportunity to ask questions and have an informed discussion. Upon oral consent to participate, written consent from patients will be obtained by SLM or UNJ.

### Assignment of interventions

Participants will be randomly assigned to either ORC or iRARC with a 1:1 allocation using the randomisation module of Research Electronical Data capture (REDCap) [17, 18]. The allocation sequence is a computer-generated list of random numbers transferred to REDCap. No stratification will be applied. Block randomisation will be used to ensure equal distribution between the two arms at any time during the trial. The block sizes will not be disclosed to ensure concealment. The web-based randomisation system ensures concealment. A collaborator with no clinical involvement has transferred the randomisation code to REDCap. Allocation concealment will be ensured as no other person has access to the randomisation code. After storing in REDCap, changing the randomisation sequence is impossible. The randomisation is performed by the Head Nurse at the Department of Urology or substitute who is the only person with access to the randomisation module in REDCap. The randomisation will be performed the day before surgery. Upon randomisation, only the staff in the operating room and the surgeons are informed. The participants, treating physicians, nurses at the PACU, and the urological ward as well as the outcome assessor are all blinded until discharge. Participants are only blinded until discharge as blinding after discharge is impossible.

### Interventions and blinding

Participants will be randomised 1:1 to receive either ORC or iRARC. All participants are treated in an ERAS setup (Table 1). Upon randomisation, only the operating surgeons, the assistant surgeon, the operating room, and anaesthesiological staff will be informed of the assigned allocation, and none of them are involved in the postoperative care. The treatment allocation is concealed from the patients, treating physicians, staff at the PACU and the urological ward, and the outcome assessor until discharge.

**Table 1:** List of components in Rigshospitalet’s Enhanced Recovery After Surgery protocol for radical cystectomy

Preoperatively	
- Pre-operative education and counselling: surgical details, hospital stay, and discharge criteria	
- Stoma education with a specialised stoma nurse	
- Preoperative medical evaluation and optimization	
- Advice and support for smoking cessation and reduction of alcohol intake	
- Instruction on postoperative mobilisation and physiotherapy	
- Anesthesiologic assessment	
Day before operation	
- Admission to hospital	
- Normal diet with no restrictions	
- Rectal enema the night before surgery; omission of mechanical bowel preparation	
- Pharmacological thrombosis prophylaxis with LMWH (Tinzaparin 4500 IE)	
POD 0: preoperatively	
- Preoperative fasting: normal diet until 6 h and clear fluids until 2 h before anaesthesia	
- Preoperative pain medication: Gabapentin 600 mg + Paracetamol 1 g.	
- No long-acting sedatives	
- Elastic compression stockings for thrombosis prophylaxis	
POD 0: intraoperatively	
- Antimicrobial prophylaxis: i.v. Cefuroxime 3 g intraoperative and skin preparation	
- Anaesthesia is similar between groups, including orotracheal intubation, total intravenous anaesthesia (Cisatracurium, Remifentanil, and Propofol), Ondansetron 4mg, Dexamethasone 24 mg, Tranexamic acid 1000mg, i.v. oxycodone and regional anaesthesia using rectus sheath blocks (2 mg bupivacaine/kg body weight) at the end of surgery without epidural	
- Central venous catheter (vena jugularis interna dexter)	
- Radial arterial line	
- Prevention of intraoperative hypothermia (Bair Hugger)	
- Nasogastric tube inserted and removed before extubating	
- No resection site drainage	
- Bilateral ureteric stents	
POD 0: postoperatively	
- Admission to the postoperative care unit until POD1	
- Chewing gum (throughout admission to hospital)	
- Antibiotics Cefuroxime 1500 mg x 3 i.v. (continued for 3 days)	
- Thrombosis prophylaxis: LMWH (continuing 4 weeks postoperatively) and compression stockings (until discharge)	
- Analgesics: Gabapentin 600mg+300 mg (continued for 3 days) + Paracetamol 1 g x 4 (continued throughout admission), *short-acting* opioids (oxycodone or morphine) if necessary	
- Laxatives: macrogol 1 sachet x 2	
- Antiemetics: Metoclopramide 10 mg if needed	
- Mobilisation: sitting and standing in the evening	
- Oral nutrition: maximum 1 L I fluid, no solids	
- Fluid strategy: Goal directed fluid therapy by stroke volume optimization	
- Continuous Positive Airway Pressure (CPAP) every second hour except during nighttime (24–06)	
POD 1	
- Admission to the urological ward	
- Medication and thrombosis prophylaxis: see “POD 0 postoperatively”	
- Oral nutrition: solids as tolerated, maximum of 1 L of fluids	
- Mobilisation: sitting as much as possible in a chair, walk minimum 2 x 60 m with a walking frame with wheels	
- Self-administration of thromboprophylaxis injections and individually adapted level of self-sufficient care for ileal conduit throughout their hospital stay in preparation for discharge	
POD 2	
- Medication and thrombosis prophylaxis: see “POD 0 postoperatively”	
- Oral nutrition: no restrictions	
- Mobilisation: out of bed minimum 2 x 3 h, walk minimum 3 x 60 m	
POD 3	
- Medication: see “POD 0 postoperatively”	
- Oral nutrition: no restrictions	
- Mobilisation: out of bed minimum 8 h, walk minimum 3 x 60 m	
- Discharge if fulfilling discharge criteria	
POD 4 and until discharge	
- Medication: Paracetamol 1 g x 4, macrogol 1 sachet x 2. Metoclopramide and short-lasting opioids if needed.	
- Mobilisation: as POD 3	
Discharge criteria	
- Adequate pain control	
- Independently mobilised	
- Instructed in stoma care, and establishment of post-discharge specialised assistance if needed - No sign of ileus	
- Adequate oral intake	
Discharge	
- Unblinding	
- Provision of support network including district nurse and urology nurse	
- Information on signs and symptoms of complications. Informed to contact the department by telephone at all hours in case of signs of complications for consultation with a urological nurse or doctor on further actions.	
Post discharge	
- Removal of ureteric stents and skin suture on POD 10 with creatinine blood sample on POD 11	
- Contact with a nurse by telephone the first Thursday after discharge	
- Follow-up after discharge will adhere to local and national guidelines with planned outpatient visit (1) 3 weeks postoperatively for the result of pathology report and planning of oncologic follow-up, and (2) 8 weeks postoperatively for a 99m Technetium-mercaptoacetyltriglycine renography control.	

*POD* postoperative day, *LMWH* low molecular weight heparin

**Fig. 1:**
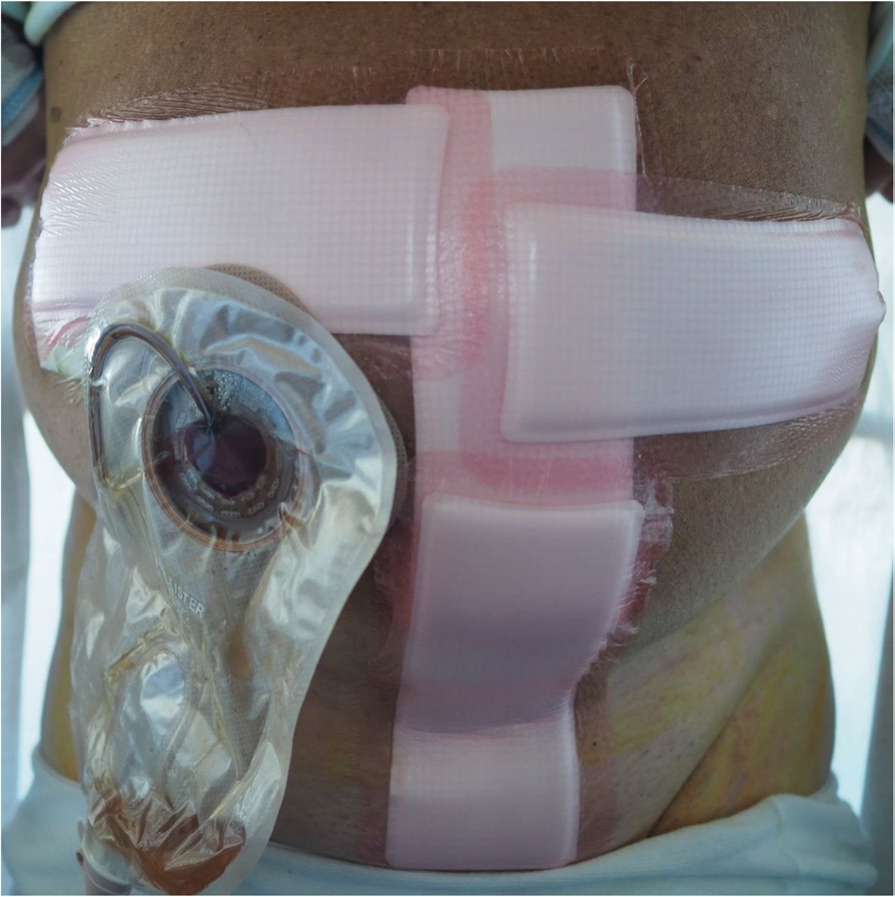
Postoperative dressing of the abdomen until discharge (picture with patient consent)

Before the start of the protocol, all study personnel have been trained in the protocol setup. The procedure will be booked as an open procedure in the electronic operating booking system and scheduled from 9 a.m. to 2 p.m. regardless of the method of surgery used. At admission, a note stating that the patient is included in this project will be placed on the patient’s bed. All personnel, including porters, are instructed not to discuss the method of the surgical procedure with the patient. All patients are operated in the same operating theatre in which the da Vinci® Surgical System robot is placed. Anaesthesia is similar between groups, including orotracheal intubation, total intravenous anaesthesia (cisatracurium, remifentanil, and propofol), i.v., oxycodone and regional anaesthesia using rectus sheath blocks (2 mg bupivacaine/kg body weight) at the end of surgery without an epidural. No pre-planning of the operating theatre is performed before the patients are sedated. Both ORC and iRARC follow the same surgical template which includes pelvic lymph node dissection and urinary diversion with an ileal conduit. The extent of the lymphadenectomy will be with the following boundaries: cranially the aortic bifurcation, laterally the genitofemoral nerves, anteriorly the pubic symphysis and the inguinal ligaments, inferiorly the pelvic floor, and posteriorly including the pre-sacral lymph nodes. All wounds are closed using non-absorbable suture. Both primary surgeons and the assisting surgeon will stay in the operating room or an adjacent room to the operating theatre throughout the entire pre-booked time period. The window in the door to the operating room will be covered by a curtain. The operating room is not located adjacent to the other urological operating rooms and no surgeon from the urological ward will pass the used operating room. A temporary standard operation report is used for all patients. In this description, important information on pathology findings and intraoperative complications are included in a manner that does not reveal the surgical method, but data on estimated blood loss (EBL) and time of surgery are left out. A detailed and final operation report will be written by the primary surgeon and kept electronically at the primary surgeon’s password-protected personal drive. The final operation report will be placed in the electronic health record (EHR) at discharge. A printed version of the temporary standard operation report will be kept in a sealed envelope in a hidden place at the Department of Urology. If necessary, this can be obtained at any time by contact with the principal investigator (PI). The abdomen will be dressed to cover wounds from both an ORC and an iRARC (Fig. 1). An adhesive non-transparent, highly absorbent dressing (i.e., Allevyn®) will be used to cover possible exuding from the wounds. The dressing is applied by the surgical team in the operating theatre. Patients are admitted to the PACU after the surgery, where similar postoperative care is performed for both groups, including goal-directed fluid therapy by cardiac output monitoring. On POD1 they are transferred to the urological ward unless the DASAIM discharge criteria are not fulfilled [19]. The dressing will not be changed unless required (because of strike-through or lack of adherence). In this case, a nurse from the neighbouring urological ward, who is not otherwise involved in the care, will be summoned. When changing the dressing, a pillow will be placed on the chest of the patient to maintain blinding of the patient. In the nurses’ office at the ward, a laminated short guide on what to do in specific situations is placed on the wall. Also, alongside this is placed a template of an abdomen with instructions on how to change and place the dressings to keep it standardised. If at any time it is assessed necessary to inspect the wound (e.g., suspicion of wound dehiscence), this can be done and documented. The dressing will be inspected for strike-through of excess exudate by a person not otherwise involved in the care of the patient at least once a day before rounds and changed if needed. SLM will ensure that participants follow the ERAS protocol. Reasons for not complying with the protocol will be registered. Patients are planned to be discharged on POD3 if fulfilling discharge criteria as outlined in Table 1.

Any deviation from the planned surgery including conversion from iRARC to ORC will be at the operating surgeon’s discretion. Postoperatively, a nasogastric tube can be inserted in case of ≥ two episodes of vomiting of an estimated volume > 200 ml. Any other prescription or change in the treatment plan will be instated and documented at the treating physician’s discretion. The patient will be discharged by the treating physician when the patient is mobilised without the need for aid, has adequate pain control and can eat and drink sufficiently. Also, unblinding is permissible at the attending physician’s discretion if examination of the wound is required for example in case of return to the operating room. All patients are planned to be unblinded at discharge unless unblinding has occurred prior. When unblinding, a report form is completed. The form includes the date and reason for unblinding the participant. The attending physician, the nurse and the participant are asked to answer their believed method of surgery: ORC, iRARC, or “do not know” and give a reason for their answer before undressing the wound.

Participants are not planned for any additional follow-up outside of the standard. No concomitant care is prohibited during the trial. Therefore, we expect non-retention in the trial to be low.

### Outcomes

#### Primary outcome

The success of blinding will be determined by (1) the number and proportion of participants with unplanned unblinding before discharge in the two treatment arms and (2) the number of participants, attending doctors, and nurses guessing the correct method of operation at unblinding beyond the level of chance. A previous study from our institution has found a reoperation rate of 22% during the first 90 days after RC with the majority being reoperated during their index hospitalisation [20]. As a reoperation necessitates unblinding, a proportion of < 25% of participants with unplanned unblinding is considered succesfull.

#### Secondary outcomes

There are 12 secondary outcomes as follows:

- LOS
- Days alive and out of hospital (DAOH)
- Conversion (from iRARC to ORC) rate
- In-hospital complication rate
- 30-day complication rate
- 90-day complication rate
- Readmission rate
- Quality of life (EORTC QLQ-C30 and EORTC QLQ-BLM30)
- Blood loss (estimated and hidden)
- Pain
- Moderate/severe complications in the PACU (DASAIM discharge criteria)
- Delirium (3-Minute Diagnostic Interview for Delirium Using the Confusion Assessment Method (3D-CAM)

#### Length of stay (LOS) and days-alive-and-out-of-hospital (DAOH)

The difference in LOS and DAOH between the two treatment arms. Both LOS and DAOH are surrogate markers for recovery after surgery. LOS is calculated as the number of days from the day of operation (POD 0) until the day of discharge and is a commonly reported outcome measure of recovery within ERAS programmes [21]. LOS is affected by local conditions as the perioperative care, discharge criteria, and the post-discharge social care setup. Also, readmissions after RC are frequent and not captured by LOS. On the contrary, DAOH is calculated as days alive and not admitted to hospital from day of operation (POD 0) to 90 days postoperatively. To calculate DAOH, the number of readmissions and the number of days readmitted will be collected.

#### Complication rate

Complications are graded using the Clavien-Dindo system and types of complications are categorised [22]. We will compare complication rates between the two treatment arms. The complication rate will be calculated as the proportion of participants who develop a complication during hospitalisation or within 30 or 90 days. A complication is defined as any deviation from the standard protocol. An unscheduled outpatient visit will be calculated as at least a grade I complication. In-hospital, 30- and 90-day complication rates are chosen as they are frequently used when reporting short-term morbidity after RC.

#### Quality of life (QoL)

To assess QoL participants will complete the validated EORTC QLQ-C30 and EORTC QLQ-BLM30 at baseline and 90-days postoperatively. EORTC QLQ-C30 has well-established reliability and validity and is frequently used in clinical trials of RC. EORTC QLQ-C30 is however not bladder specific. Therefore, QLQ-BLM30 will be used as an addition. This questionnaire has been used in several publications before but is not validated. Since most studies use the EORTC QLQ-C30 and QLQ-BLM30 comparative integration of the results in the existing literature will be easy. Patient-reported outcomes are reported according to standards recommended by the 2010 Consolidated Standards of Reporting Trials (CONSORT) PRO extension [23].

#### Blood loss

Formulas exist for estimating blood loss using blood samples (haemoglobin), number of transfusions, and estimated blood volume [24]. We will collect both EBL as well as measures for estimating blood loss by a formula.

#### Pain

The difference in daily visual analogue scale and consumed daily and the total amount of opioids will be compared between the two treatment arms.

#### Post-anaesthesia care unit complications

The difference in rate of moderate/severe complications in the PACU and time from surgery to development of a moderate/severe complication in the PACU. A moderate/severe complication is defined as a score of > 1 according to the discharge criteria scoring system recommended by DASAIM [19].

#### Delirium

The occurrence of acute postoperative delirium is assessed by the Danish version of the 3D-CAM on POD 0 and POD 1 before discharge to the urological ward.

A schedule of enrolment, intervention, and assessments is depicted in Table 2.

**Table 2:** Participant timeline

	Preoperative		Perioperatively (RC)	Postoperatively		
Assessment	Outpatient visit	Baseline	POD 0	POD 0 - discharge	Discharge – POD 90	POD 90
Enrolment						
Eligibility screen	x					
Informed consent	x					
Allocation		x				
Medical history, demographic data		x				
Physical examination incl. blood pressure, weight		x		x		
MMSE		x				
Questionnaires						
EORTC QLQ-C30		x				x
EORTC QLQ-BLM30		x				x
Paraclinical examinations:						
Blood samples		x	x	x		
Intraoperative data					x	
3D-CAM				x^*^		
Daily morphine use				x		
Reason(s) for not being discharged				x		
Clavien-Dindo assessment				x	x	x
Adverse events			x	x	x	x
LOS and DAOH						x
Blood loss					x	

*POD* postoperative day, *MMSE* Mini-Mental State Examination, *EORTC* European Organisation for Research and Treatment of Cancer, *3D-CAM* 3-Minute Diagnostic Interview for Delirium Using the Confusion Assessment Method, *LOS* length of stay, *DAOH* days alive and out of hospital

^*^Only assessed POD 0 and POD 1

### Data collection and management

All data are entered in data collection instruments in REDCap. REDCap provides features to maintain data quality. All variables are enforced a validation standard on the field. Validation prevents users from entering data in an incorrect format, as saving the data is impossible if data fails to match the validation format. Range checks are applied on all number fields so REDCap will warn the user if an entered value is out of the expected range. Also, some data collection instruments have validated branching logic and/or calculations built in. Warnings are given by REDCap when leaving a data collection instrument if not all data fields are filled in to prevent missing data. After completion of the study, the data will be stored in REDCap until 31st of January 2029.

The calendar function in REDCap is used to create reminders for contacting participants 90 days postoperatively. This is to ensure that any complication or adverse events handled out of the hospital is captured and to ensure participants to answer QLQ. The eCRF is stored in a REDCap electronic data capture tools hosted at the Capital Region of Denmark. The database is password-protected and only accessible to assigned persons. All participants are given a unique study identification number to extract pseudo-anonymized data from REDCap to statistical software programmes. Furthermore, identifying information on participants in REDCap will be marked by a “participant identifier” and this information cannot be extracted from the database. Written consent forms are stored in a locked filing cabinet.

### Statistics

For the primary outcome, the proportion of participants with unplanned unblinding before discharge in the two treatment arms will be calculated using descriptive statictics. The number of participants guessing the correct method of operation at unblinding beyond the level of chance is calculated using Bang’s Blinding Index [25].

Analyses and data summaries will be conducted using the groups to which the patients were randomised, and patients will be included regardless of whether they received the allocated treatment, or they were unblinded during admission (“intention to treat”). Baseline characteristics of the included patients will be summarised by trial arm using descriptive statistics with continuous variables reported as the median and interquartile range (IQR) and categorical values in numbers and percentages of the total group. Full details will be described in the statistical analysis plan for the study.

We expect the amount of missing data to be low as most data is extracted from the EHR. We also expect dropouts to be low given the non-experimental design of the study with no extra planned physical visits out of the ordinary follow-up. In the case of dropouts, we expect these to be “missing not at random.” Therefore, multiple imputation will be used for handling missing data.

The protocol, template for the eCRF including the data collection instruments, de-identified data, and the statistical code are not made publicly available but will be delivered upon reasonable request by contacting the PI or the corresponding author. However, access to de-identified data will require an application to and approval from the Danish Data Protection Agency.

### Monitoring

As this is a single-centre feasibility study no coordination centre or various committees are planned. The PI, SLM, EKA, MAR, and HK have been in constant contact and have had arranged meetings approximately every third month to discuss progress, data quality, and potential challenges in the conduction of the trial. No data monitoring committee is planned due to the exploratory nature of the study design. Furthermore, the risk profile of the trial design is assessed to be minimal. The PI will make safety and progress reports to the ethics committee at least annually and within 90 days of study termination or completion. In case of serious adverse events beyond what can be expected from RC, the ethics committee will be notified immediately, and no longer than 2 weeks after.

### Ethics and dissemination plans

The Danish Scientific Ethical Committee System (journal number: H-18056682) has approved the trial. The PI will be responsible for any amendments to the protocol. Any modifications will be reviewed and approved by the Danish Regional Ethics Committee. In this case, the protocol name will be changed so the suffix will indicate the version number and new date (e.g., v2_ddmmyy, v3_ddmmyy). A list of amendments will be created to keep track.

Both ORC and iRARC in a modified ERAS protocol are offered as standard treatments at the Department of Urology, Rigshospitalet, and is provided for by the Danish healthcare system. No participants are subjected to any kind of experimental treatment. Therefore, we do not expect any harm from the trial. All areas in the Danish healthcare system are covered by a publicly funded compensation scheme. All participants can file a claim for an injury sustained as a result of the treatment through the Patient Compensation Association as everyone else who receives treatment or purchases medicine in Denmark.

The trial results will be published in peer-reviewed international journals or otherwise made publicly available and will be presented at national and international conferences and symposiums irrespective of the outcomes. In a 30- and 90-day complication rates, QoL and PACU complications will be reported in separate publications. Study completion is expected by March 2021, and dissemination of the results will begin as soon as possible thereafter.

## Discussion

RC remains among the most complex urological procedures with a high risk of short- and long-term morbidity and mortality. Advancements in technology, urologic and anaesthesiologic care, and structured protocols for enhanced surgical recovery have aimed to reduce complications after RC. In the past decade, the evolution of surgical robots has fueled the interest for laparoscopic surgery and has encouraged the surgical community to anticipate that robot-assisted laparoscopic surgery is superior to open surgery, especially in terms of complications and recovery. However, with both patient safety and increasing expenses in health care in mind, it is important to be critical when introducing new medical equipment and surgical techniques. Surgical trials are difficult to successfully undertake and pose specific practical and methodological challenges [26]. The IDEAL collaboration has formed a framework for the assessment of surgery based on a five-stage description of the surgical development process [27]. These recommendations offer good practical guidance. Unlike areas of pharmaceutical interventions, the evaluation of surgical interventions with RCTs is not mandatory, and most likely, the absence of a regulatory framework has contributed to the proliferation of surgical innovation based on limited and weak scientific evidence.

To date, four RCTs have compared ORC to RARC [5–8]. Nix et al. reported the first trial of ORC versus RARC in 2010 [5]. It was a non-inferiority trial investigating the lymph node yield between the two procedures and demonstrated that RARC was non-inferior to ORC. The study also showed a significant difference in EBL, time to flatus, time to bowel movement, and use of inpatient morphine sulphate equivalents in favour of RARC although operative time favoured ORC. The study found no significant difference in the overall complication rate or LOS. Both Bochner et al. in 2015 and Khan et al. in 2016 investigated ORC versus RARC with complications as the primary outcomes [6, 8]. Bochner et al. reported the overall 90-day grade II–V Clavien-Dindo complications as primary outcome and found no significant difference between ORC and RARC [6]. Results from secondary outcomes included a significant difference in operative time and costs in favour of ORC and EBL in favour of RARC. No significant difference was found in high-grade complications, pathological outcomes, or QoL between the two arms. Oncological outcomes such as the risk of recurrence, patterns of recurrence, and survival did not differ between ORC and RARC [28]. Khan et al. compared laparoscopic RC, RARC, and ORC for complications (Clavien-Dindo) at 30 and 90 days after surgery and found no difference in complication rates between ORC and RARC. Operative time favoured ORC, but no significant difference was found in other secondary outcomes. The most recently published trial of ORC versus RARC is the RAZOR trial [7]. It was a non-inferiority trial with progression-free survival at 2 years as the primary outcome demonstrating RARC to be non-inferior to ORC. Of significant secondary outcomes, they found a difference in EBL, postoperative blood transfusion, and LOS in favour of RARC and a significant difference in operative time in favour of ORC. No significant difference was found in overall complication rate, major complication rate or QoL.

In the previous RCTs, the urinary diversion for RARC has been performed extracorporeally. A completely intracorporeal procedure could potentially hold advantages over ORC. This is currently being investigated in the ongoing iROC trial which is designed to show a difference in recovery in terms of DAOH [29]. However, this trial is not blinded. The lack of blinding may cause bias, especially if the level of subjectivity in the outcome variable is low. In trials comparing laparoscopic and mini-laparotomy cholecystectomy, non-blinded RCTs found a shorter recovery in terms of LOS and time back to work with the laparoscopic technique [30, 31]. However, in a blinded RCT of laparoscopic versus mini-laparotomy cholecystectomy no difference in outcomes was found [32].

None of the previously conducted studies has the perioperative care been described as standardised in an ERAS setup. ERAS focus on promoting recovery by reducing the surgical stress response and postoperative organ dysfunction. We believe a blinded trial with a strict ERAS protocol in RC is necessary to further understand the true differences between RARC and ORC.

The success of the blinding in our study is dependent on two aspects. Firstly, the amount of “intentional” unblinding during admission to the hospital would reveal the method of surgery. Secondly, “unintentional” unblinding can happen if variables that reveal the used procedure are not masked. From previous RCTs, a significantly lower EBL is known with RARC and a shorter operative time with ORC [5–9]. Also, the surgical wound on the abdomen will reveal the used procedure. These factors might affect how mobilisation is advised, painkillers are administered, etc. It may also affect the patients’ experience and expectations for post-surgical care. Therefore, blinding in RC patients may reduce the many biases introduced in the previous RCTs. But blinding of surgical procedures is challenging at both patient and physician level. However, in abdominal surgery, it has been shown that blinding is feasible by covering the whole abdomen with a bandage [11–14]. In RC, the dressing also needs to consider the urinary stoma which poses a technical challenge. We solved this by designing a dressing, together with experienced nurses, which was tested before study initiation. We have also made detailed instructions for changing the dressing including a template for how to place the dressing. To maintain blinding, a nurse from the neighbouring ward is summoned if the dressing needs to be changed and a pillow will be placed on the patient’s chest to block their view of the wound. Patient instruction and practice in changing of the stoma appliance can be carried out as needed as the stoma is free of the customised dressing.

In short, this protocol describes the first trial that evaluates the feasibility of a blinded study comparing ORC and RARC in an ERAS setup. Evidence from studies of high methodological quality is much needed in the field of RC. Lessons learned from this trial could prove valuable for the design and execution of future trials.

### Trial status

The original first version from 14th of December 2018: Protocol-60292_v1_141218. The study was initiated in June 2019, and 47 participants have been enrolled so far. We expect completion of participant accrual in December 2020.

## Supplementary Information

**Additional file 1.**

**Additional file 2.**

**Additional file 3.**

## Data Availability

After study completion and publication of the study, the dataset analysed during the current study will be available from the corresponding author on reasonable request and upon approval from the Danish Data Protection Agency.

## Abbreviations

RC: Radical cystectomy
BC: Bladder cancer
ORC: Open radical cystectomy
RARC: Robot-assisted laparoscopic radical cystectomy
iRARC: Robot-assisted laparoscopic radical cystectomy with intracorporeal urinary diversion
RCT: Randomised clinical trial
LOS: Length of stay
DAOH: Days-alive-and-out-of-hospital
EORTC: European Organisation for Research and Treatment of Cancer
QLQ: Quality of life questionnaire
PACU: Post-anaesthesia care unit
EBL: Estimated blood loss
EHR: Electronic health record
PI: Principal investigator
DASAIM: Danish Society of Anaesthesiology and Intensive Care Medicine
3D-CAM: 3-Minute Diagnostic Interview for Delirium Using the Confusion Assessment Method
eCRF: Electronic case-report form
