# Supplementary material for: Robot-assisted laparoscopic radical cystectomy with intracorporeal ileal conduit diversion versus open radical cystectomy with ileal conduit for bladder cancer in an ERAS setup (BORARC): protocol for a single-centre, double-blinded, randomised feasibility study
Source: Pilot Feasibility Stud. 2023 Jan 13;9:7. doi: 10.1186/s40814-022-01229-3 (PMC9838067; doi:10.1186/s40814-022-01229-3)
Supplement: Supplementary file 1 — Additional file 1. [file 40814_2022_1229_MOESM1_ESM.pdf]

Informeret samtykke til deltagelse i forskningsprojekt- Samtykkeerklæring

Forskningsprojektets titel: Radikal cystektomi med åben eller robotassisteret teknik: et blindet, randomiseret, kontrolleret studie.

Erklæring fra deltageren

Jeg har fået skriftlig og mundtlig information om projektet, og jeg ved nok om formål, metode, fordele og ulemper til at sige ja til at deltage.

Jeg ved, at det er frivilligt at deltage, og at jeg altid kan trække mit samtykke tilbage uden at skulle give en forklaring.

Jeg ønsker at deltage i forskningsprojektet og har fået kopi af dette samtykkeark samt en kopi af den skriftlige information om projektet til eget brug.

Projektdeltagerens navn:

Dato: Underskrift:

Jeg ønsker at blive informeret om forskningsprojektets resultater (sæt X):

Ja ☐ Nej ☐

Jeg giver tilladelse til, at jeg må kontaktes med information om deltagelse i senere faser af projektet, hvis det er relevant (sæt X):

Ja ☐ Nej ☐

Hvis "ja" til et af ovenstående spørgsmål, da angives e-mail adresse eller postadresse, hvor du ønskes kontaktet:

Erklæring fra den informerende

Jeg erklærer, at deltageren har modtaget mundtlig og skriftlig information om forskningsprojektet og har haft mulighed for at stille spørgsmål til mig.

Efter min overbevisning er der givet fyldestgørende information således, at der kan træffes beslutning om deltagelse i forskningsprojektet.

Den informerendes navn:

Dato: Underskrift:
