# Supplementary material for: Robot-assisted laparoscopic radical cystectomy with intracorporeal ileal conduit diversion versus open radical cystectomy with ileal conduit for bladder cancer in an ERAS setup (BORARC): protocol for a single-centre, double-blinded, randomised feasibility study
Source: Pilot Feasibility Stud. 2023 Jan 13;9:7. doi: 10.1186/s40814-022-01229-3 (PMC9838067; doi:10.1186/s40814-022-01229-3)
Supplement: Supplementary file 2 — Additional file 2. [file 40814_2022_1229_MOESM2_ESM.pdf]

Deltagerinformation om et videnskabeligt forsøg.

Forsøgets titel: Radikal cystektomi med åben eller robotassisteret teknik: et blindet, randomiseret, kontrolleret studie.

Vi vil spørge, om du vil deltage i et videnskabeligt forsøg, der udføres af Urologisk Klinik, Rigshospitalet i samarbejde med Anæstesiologisk Klinik og Enhed for Kirurgisk Patofysiologi, Rigshospitalet. Urologisk Klinik er ansvarlig for kirurgisk behandling på urinvejene, Anæstesiologisk afdeling er ansvarlige for bedøvelse og varetagelse af patienter under operation og Enhed for Kirurgisk Patofysiologi igangsætter og koordinerer aktiviteter til forbedring af de kirurgiske indgreb både før, under og efter en operation.

Før du beslutter, om du vil deltage i forsøget, skal du fuldt ud forstå, hvad forsøget går ud på, og hvorfor vi gennemfører forsøget. Vi vil derfor bede dig om at læse denne deltagerinformation grundigt.

Du vil blive tilbudt en samtale om forsøget, hvor denne deltagerinformation vil blive uddybet, og hvor du kan stille de spørgsmål, du har om forsøget. Du er velkommen til at tage et familiemedlem, en ven eller en bekendt med til samtalen. Hvis du beslutter dig for at deltage i forsøget, vil vi bede dig om at underskrive en samtykkeerklæring. Husk, at du har ret til betænkningstid, før du beslutter, om du vil underskrive samtykkeerklæringen.

Det er frivilligt, om du vil deltage, og såfremt du ikke ønsker at deltage, vil dit valg ikke påvirke din nuværende eller fremtidige behandling. Hvis du vælger at deltage og senere fortryder, kan du altid med øjeblikkelig virkning trække dit tilsagn tilbage, uden at du behøver at give en begrundelse.

Formål med forsøget

Operation for blærekræft (cystektomi) indebærer fjernelse af urinblæren og lymfeknuder i bækkenet samt urinafledning med brug af et tarmstykke til en urostomi. Operationen kan enten foretages som en åben operation eller som en robotassisteret kikkert-operation. Det er vigtigt for os at understrege, at begge operationstyper tilbydes som ligeværdige standardbehandlinger på Rigshospitalet aktuelt. Vi ønsker i dette forsøg, at sammenligne de to operationsteknikker for at undersøge, om der er fordele ved den ene metode i forhold til den anden.

Metode

Vi ønsker at sammenligne operationsmetoderne hos i alt 50 patienter, der ved tilfældig tildeling (randomisering) vil blive opereret enten med robotassisteret kikkertoperation eller ved åben operation. Dette vil foregå uden, at man som patient på forhånd er vidende om med hvilken teknik, man opereres. Alle patienter opereres af en erfaren primærkirurg enten overlæge Peter Thind, der opererer åbent eller overlæge Malene Rohrsted, der opererer robotassisteret. En af afdelingens øvrige læger vil assistere og hjælpe ved operationen. Efter operationen vil såret blive tildækket med et plaster, der dækker hele maven, så det ikke afsløres med hvilken teknik, man er opereret. Hverken du som patient, sygeplejersker eller de læger som går stuegang efter operationen, vil være bekendt med operationsmetoden. Det er nødvendigt, at dette er skjult, da forventninger til en operationsmetode kan have indvirkning på behandlingen og dermed resultaterne.

Ved udskrivelsen bliver forbindingen taget af og du vil blive informeret om, hvilken metode, du er opereret med. Såfremt der skulle opstå komplikationer i forbindelse med indlæggelsen, hvor det lægeligt skønnes nødvendigt at aftage forbindingen, vil dette selvfølgelig blive gjort.

Plan for forsøget
Forsøget starter den dag, du indvilger i at deltage og afsluttes 90 dage efter operationen, hvorefter du fortsat vil blive fulgt i afdelingen i henhold til de sædvanlige retningslinjer for opfølgning af patienter, som er blevet opereret for kræft i blæren.

Udover de sædvanlige aftaler i forbindelse med en operation for kræft i blæren, vil du som forsøgsdeltager skulle gennemgå følgende:

- udfyldelse af to spørgeskemaer før operationen og 90 dage efter operationen

Bivirkninger, risici, komplikationer og ulemper
Bivirkninger, risici og komplikationer er som ved standardbehandlingen for kræft i blæren. Der er ingen eksperimentel behandling. Ulemperne er udover de ekstraundersøgelser som ovenfor beskrevet det faktum, at du ikke før og umiddelbart efter operationen vil vide med hvilken operationsteknik, du er blevet opereret med samt, at du efterfølgende har en større bandage på maven end vi vanligt forbinder med. Forsøget indebærer ikke ekstra besøg i klinikken eller ekstra prøver af nogen art forbundet med ubehag.

Andre behandlingsmuligheder
Såfremt du ikke ønsker at deltage i forsøget vil du blive tilbudt enten robotassisteret eller åben operation efter vanlig procedure. Tilbuddet om åben eller robotassisteret operation afhænger af ledig kapacitet på vores operationsgang, og vi kan ikke altid tilbyde frit valg mellem operationsformerne indenfor behandlingsgarantien.

Journaloplysninger
Vi vil indhente oplysninger fra din journal, for at se om der er forskel på de patienter, der får tildelt de to operationstyper, og for at sammenligne hvordan det går i perioden efter operationen. Vi indhenter oplysninger om de blodprøver, som alligevel tages under operationen og de observationer som gøres under din indlæggelse (f.eks. dine symptomer, blodtryk, temperatur) samt eventuelle komplikationer som står i journalen af det behandlende personale. Samtykke til forsøget giver de forsøgsansvarlige direkte adgang til relevante helbredsoplysninger i journalen for at kunne gennemføre, overvåge og kontrollere forsøget.

Nytte ved forsøget
Deltagende patienter kan medvirke til at afklare mulige forskelle afhængig af operationsteknik i forløbet under og efter operation hos patienter, der får foretaget fjernelse af urinblære og lymfeknuder i bækkenet samt anlagt urinafledning. Studiet kan herved være med til at afklare, om robotassisteret kikkertoperation tilbyder fordele eller ulemper frem for åben operation. Der vil ikke som udgangspunkt være nogen personlig fordel for den enkelte patient ved deltagelse.

Udelukkelse fra og afbrydelse af forsøg

Flere situationer kan føre til, at du tages ud af forsøget:

- Såfremt du ikke længere ønsker at deltage.
- Såfremt du bliver opereret robotassisteret og det er nødvendigt at ændre operationen til en åben operation.
- Såfremt bandagen fjernes inden udskrivelse.
- Såfremt nogle af forsøgsreglerne ikke bliver overholdt.
-

Økonomiske forhold

Urologisk Klinik, Rigshospitalet har taget initiativ til forsøget og afholder størstedelen af udgifterne forbundet hermed. Der vil desuden blive søgt økonomisk støtte til projektet via private og/eller offentlige fonde. Når den endelige finansiering foreligger vil Videnskabsetisk Komite blive orienteret og protokol samt deltagerinformation vil blive opdateret med de nye oplysninger. Der vil ikke blive tilbudt vederlag for deltagelse i projektet.

Adgang til forsøgsresultater

Når forsøget er afsluttet vil resultaterne blive udgivet i et internationalt tidsskrift. Vi forventer, at forsøget vil blive afsluttet i løbet af 2020, og at resultater vil blive opgjort og offentliggjort herefter.

Projektgodkendelse, monitorering og databehandling

Studiet er godkendt af Datatilsynet og Den Videnskabsetiske Komité. Alle forsøgsdeltageres data beskyttes efter databebeskyttelsesloven/persondataforordningen og sundhedsloven. Såvel læger som de involverede myndigheder er underlagt tavshedspligt.

Vi håber, at du med denne information har fået tilstrækkeligt indblik i, hvad det vil sige at deltage i forsøget, og at du føler dig rustet til at tage beslutningen om din eventuelle deltagelse. Vi beder dig også om at læse det vedlagte materiale "Forsøgspersoners rettigheder i et sundhedsvidenskabeligt forskningsprojekt".

Hvis du vil vide mere om forsøget, er du meget velkommen til at kontakte os.

Med venlig hilsen,
på vegne af urologisk afdeling

Sophia Maibom, læge
Ph.d.-studerende/klinisk assistent
Rigshospitalet
Urologisk Forskningsenhed (afsnit 7521)
Ole Maaløes Vej 24
2200 København N
e-mail: sophia.liff.maibom@regionh.dk
Telefon 35 45 61 53

Ulla Nordström Joensen, afdelingslæge
Forsøgsansvarlig
Rigshospitalet
Urologisk Klinik (afsnit 2111)
Blegdamsvej 9
2100 København Ø
e-mail: ulla.nordstroem.joensen@regionh.dk
