# Supplementary material for: Robot-assisted laparoscopic radical cystectomy with intracorporeal ileal conduit diversion versus open radical cystectomy with ileal conduit for bladder cancer in an ERAS setup (BORARC): protocol for a single-centre, double-blinded, randomised feasibility study
Source: Pilot Feasibility Stud. 2023 Jan 13;9:7. doi: 10.1186/s40814-022-01229-3 (PMC9838067; doi:10.1186/s40814-022-01229-3)
Supplement: Supplementary file 3 — Additional file 3. [file 40814_2022_1229_MOESM3_ESM.pdf]

Forsøgspersoners rettigheder i et sundhedsvidenskabeligt

Forskningsprojekt

Som deltager i et sundhedsvidenskabeligt forskningsprojekt skal du vide, at:

- din deltagelse i forskningsprojektet er helt frivillig og kun kan ske efter, at du har fået både skriftlig og mundtlig information om forskningsprojektet og underskrevet samtykkeerklæringen.

- du til enhver tid mundtligt, skriftligt eller ved anden klar tilkendegivelse kan trække dit samtykke til deltagelse tilbage og udtræde af forskningsprojektet. Såfremt du trækker dit samtykke tilbage påvirker dette ikke din ret til nuværende eller fremtidig behandling eller andre rettigheder, som du måtte have.

- du har ret til at tage et familiemedlem, en ven eller en bekendt med til informationssamtalen.

- du har ret til betænkningstid, før du underskriver samtykkeerklæringen.

- oplysninger om dine helbredsforhold, øvrige rent private forhold og andre fortrolige oplysninger om dig, som fremkommer i forbindelse med forskningsprojektet, er omfattet af tavshedspligt.

- opbevaring af oplysninger om dig, herunder oplysninger i dine blodprøver og væv, sker efter reglerne i databeskyttelsesforordningen, databeskyttelsesloven samt sundhedsloven.

- der er mulighed for at få aktindsigt i forsøgsprotokoller efter offentlighedslovens bestemmelser. Det vil sige, at du kan få adgang til at se alle papirer vedrørende din deltagelse i forsøget, bortset fra de dele, som indeholder forretningshemmeligheder eller fortrolige oplysninger om andre.

- der er mulighed for at klage og få erstatning efter reglerne i lov om klage- og erstatningsadgang inden for sundhedsvæsenet. Hvis der under forsøget skulle opstå en skade kan du henvende dig til Patienterstatningen, se nærmere på www.patienterstatningen.dk.

De Videnskabsetiske Komiteer for Region Hovedstaden (6 komiteer)
Tlf.: +45 38 66 63 95
E-mail: vek@regionh.dk
Hjemmeside: www.regionh.dk/vek

Den Videnskabsetiske Komité for Region Sjælland
Tlf.: +45 93 56 60 00
E-mail: RVK-sjaelland@regionsjaelland.dk
Hjemmeside: www.regionsjaelland.dk/sundhed/forskning/forfagfolk/videnskabsetisk-komite/Sider/default.aspx

De Videnskabsetiske Komiteer for Region Syddanmark (2 komiteer)
Tlf.: + 45 76 63 82 21
E-mail: komite@rsyd.dk
Hjemmeside: www.regionsyddanmark.dk/komite

De Videnskabsetiske Komiteer for Region Midtjylland (2 komiteer)
Tlf.: +45 78 41 01 83
/ +45 78 41 01 82 / +45 78 41 01 81
E-mail: komite@rm.dk
Hjemmeside: www.komite.rm.dk

Den Videnskabsetiske Komité for Region Nordjylland Tlf.:
+45 97 64 84 40
E-mail: vek@rn.dk
Hjemmeside: www.vek.rn.dk

National Videnskabsetisk Komité
Tlf.: +45 72 21 68 55
E-mail: kontakt@nvk.dk
Hjemmeside: www.nvk.dk

Dette tillæg er udarbejdet af det videnskabsetiske komitésystem og kan vedhæftes den skriftlige information om det sundhedsvidenskabelige forskningsprojekt. Spørgsmål til et konkret projekt skal rettes til projektets forsøgsansvarlige. Generelle spørgsmål til forsøgspersoners rettigheder kan rettes til den komité, som har godkendt projektet.
